# Treating Children With Speech Sound Disorders: Development of a Tangible Artefact Prototype

**DOI:** 10.2196/13861

**Published:** 2019-12-05

**Authors:** Joaquim Santos, Mário Vairinhos, Luis M T Jesus

**Affiliations:** 1 Escola Superior de Saúde (ESSUA) Universidade de Aveiro Campus Universitário de Santiago Aveiro Portugal

**Keywords:** children, tangible artefact, speech sound disorders, exploratory test

## Abstract

**Background:**

A prototype of a tangible user interface (TUI) for a fishing game, which is intended to be used by children with speech sound disorders (SSD), speech and language therapists (SLTs), and kindergarten teachers and assistants (KTAs) and parents alike, has been developed and tested.

**Objective:**

The aim of this study was to answer the following question: How can TUIs be used as a tool to help in interventions for children with SSD?

**Methods:**

To obtain feedback and to ensure that the prototype was being developed according to the needs of the identified target users, an exploratory test was prepared and carried out. During this test using an ethnographic approach, an observation grid, a semistructured questionnaire, and interviews were used to gather data. A total of 4 different types of stakeholders (sample size of 10) tested the prototype: 2 SLTs, 2 KTAs, and 6 children.

**Results:**

The analysis of quantitative and qualitative data revealed that the prototype addresses the existing needs of SLTs and KTAs, and it revealed that 5 out of 6 (83%) children enjoyed the activity. Results also revealed a high replay value, with all children saying they would play more.

**Conclusions:**

Serious games and tangible interaction for learning and problem solving serve both teachers and children, as children enjoy playing, and, through a playful approach, learning is facilitated. A clear pattern was observed: Children enjoyed playing, and numerous valid indicators showed the transposition of the traditional game into the TUI artefact was successful. The game is varied and rich enough to be attractive and fun. There is a clear need and interest in similar objects from SLTs and educators. However, the process should be even more iterative, with a multidisciplinary team, and all end users should be able to participate as co-designers.

## Introduction

The prevalence of speech sound disorders (SSD) in the United Kingdom is estimated to range from 2% to 25% in children aged 5 to 7 years [1]. In Portugal, where the fieldwork reported in this paper took place, it is estimated that thousands of preschool aged children (8%-11% of the total population) need speech and language therapy [2]. However, owing to budget cuts, schools have fewer professionals to intervene; therefore, the role of parents and kindergarten teachers and assistants (KTAs) is particularly important [3], as the significant effects that SSD can have later in life are well documented [4-6].

Speech and language therapists use physical media (games and assorted toys) to stimulate speech and help children to overcome SSD [7]. A physical material has intrinsic qualities, such as weight, form, smell, or texture, and these can be used to stimulate speech production, thus supporting SLT interventions. A digital app, especially if tactile or mobile based, has the appeal of being a well-known device by many children, and this produces a sense of engagement through the use of sound, animation, and color. A multimodal approach, although in need of further research, appears to be effective in several fields of speech therapy [8,9], as well as other areas [10]. What is described in this paper is the creation of a hybrid artifact, capable of combining physical and digital media—multimodal, being used by speech and language therapists (SLTs), KTAs, parents and children in a one-on-one session or as group activity, with both children with SSD and typically developing children.

In this paper, the theoretical background will be discussed, as well as previous relevant projects and their contributions to the development of the current prototype; the methodology and the fishing game tangible interface (FGTI) prototype, its functional design, and technical requirements; the prototype development phase (ie, the parts that make the prototype and its iterations); detailed results of the exploratory test. Conclusions and future work are presented in the final section. The aim of this paper is to determine how can tangible user interfaces (TUIs) be used as a tool to help in interventions for children with SSD?

### Speech Sound Disorders

SSD take the form of gaps in children’s speech sound systems, which can cause difficulties in producing or understanding phonemes [7]. Children with SSDs also exhibit speech patterns and structures that should not be present in typically developing children of their age [7]. A child might use, on a regular basis, what is designated by SLTs as a phonological process, for example, *final consonant deletion* (ie, the child omits a consonant in the final position of the syllable or final position in the word) [11]: The Portuguese word *<porco>* (in English, <pig>) is produced as *<poco>*.

### Role of Parents and Kindergarten Teachers and Assistants’ Roles

The current recommended speech and language therapy practices point to a family-centered intervention, promoting not only the parents’ involvement in the sessions and in homework activities but also in planning a session and setting goals. Family-centered guidelines integrate the whole family as a client, positive family and professional relationships, parental decision making, and the empowerment and enablement of families [12].

KTAs are of great importance to child development because of the time they spend with children and the nature of their relationship. They are part of a child’s innermost circle [10] and can help in the detection and reporting of possible cases of SSD, as well as in the implementation of specific activities with a child, as long as proper training, support, and tools are provided to them by SLTs. KTAs are well aware of the cognitive and social impacts of SSD in children and the negative attitudes people tend to have toward them [13]. However, a caregiver must attend the needs of several children, and in Portugal, activities have to be group based and have to benefit all.

### Tangible Artefacts

Beyond conventional interaction paradigms, such as graphical user interfaces or command line interfaces, there are several interaction paradigms (eg, natural interaction, ubiquitous computing, pervasive computing, mixed realities, or wearable computing) that can incorporate the activity context in an effortless interaction approach. The role that tangible user interfaces (TUIs) can play in education and health, the concept of interaction and how it differs from adults to children, and some psychological aspects that affect how children learn are briefly discussed in this paper.

### Tangible User Interfaces

TUIs seek to move away from the generic combination of screen, mouse, and keyboard interaction and attempt to transform the world itself into an interface [14]. They can be defined as interfaces that support users’ direct interaction with the digital world or digital device by use of real-world objects or tools [15]. They use physical forms designed and improved over millennia to fit a specific task [16], facilitating the user’s discernibility and direct manipulation of the interface through the user's peripheral senses (eg, touch or vision), because of its physical embodiment [14,16]. The user can focus his or her attention and consciousness on the task and not on the interface [17]. According to Norman [18], people develop throughout their lives a process of uninterrupted adaptation to and with the environment and an understanding of how to act in the physical world. It is from this seemingly innate understanding from which the concept of *affordances* stems. Affordances, according to the original definition by Gibson [19]—particularly the affordances of the environment—are what the environment offers the animal, whether for its welfare or unease. The affordances theory lies at the center of the conceptual model of TUIs, as the incorporation of digital technology into objects of the physical world will make the interface more familiar and easier to understand from the user’s viewpoint. TUIs can be approached as rigid discrete interfaces that use certain objects or shapes with which the user would interact and which have a perceived meaning with a finite set of objects and possible interactions [16], or they can be perceived as a more “organic” and material malleable, taking advantage of new digital and physical materials that can seamlessly pair sensing and display capabilities. These interfaces have the potential to break the boundaries of predetermined interactions [16].

#### Tangible Artefacts in Education and Health

Tangible artefacts have long been used in interactive games in therapeutic contexts, especially in fields of cerebral palsy or poststroke recovery [20,21]. They also allow one to assess several physiological parameters, without any stress associated with a visit to a doctor’s office, relieving an anxiety felt by many children and some adults alike [21].

In education, both the needs of the teachers and the needs and curricula of the students have to be fully understood and satisfied [22]. Serious games and tangible interaction for learning and problem solving serve both teachers and children. Children enjoy playing, and, through a playful approach, learning is facilitated [23]. Tangible artefacts by nature invite collaboration, allowing several users to interact with the artefact and themselves [23], thus increasing productivity levels [21], particularly as TUIs provide an interface that is space multiplexed instead of the time-multiplexed interaction, as we can typically find on conventional digital interfaces that rely on mouse and keyboard. The way in which the user interacts has to be driven through affordances, mappings, and game logic to ensure reliability and take full advantage of the potential of the artifact [21,24].

#### Designing Interaction for and With Children

Designing for interaction is all about how to design for people, their needs, emotions, and intellect, making it imperative to be highly aware of what to expect from those who will interact with the final product [25]. With the shift toward participatory and ethnographic methods, those designing interactive apps or objects have to fully understand how and why people use technological innovation [22,25].

The children (aged 3-7 years) targeted with the artefact created during this project are preliterate. With short attention spans, they have difficulty conceiving abstractions, and their fine motor skills are not yet fully developed [22,26]. Nonetheless, designing in a way that can be perceived as too childish can be felt to be boring or disrespectful by the children [22], as they are acutely aware of their capabilities [27]. A workaround is to embrace designing with children as co-designers, evaluators or subjects, or a combination of these. This approach has its own drawbacks, requiring that adults and children must work together, but in the end, this method assures that the design meets the needs and specificities of children [22].

#### How Do We Learn?

Children search for multimodal stimulation, which consequently encourages their physical and cognitive development. They are naturally motivated to explore what is around them by engaging with their environment, their medium and substance, which consequently affords certain immediate perceived venues of action, manufacture, and manipulation [19], reinforcing learning through the dynamics of play.

Cultural and social contexts influence how children construct the world and their knowledge [28]. Social experience is a critical factor in mental development [29]—interpersonal connections and social interaction provide the means for a child to access experiences that they can then integrate into their view of how things work [28]. Play helps a child to separate the meaning of an object from the actual object, and from a child’s point of view, it is not just a game, it is a serious thing, which they consider as work [28]. Adults, who make up a large part of our stakeholders, also benefit from something playful and fun. As Donald Norman says in Emotional Design: Why We Love or Hate Everyday Things [30] (page 103), “Beauty, fun, and pleasure all work together to produce enjoyment, a state of positive affect.” These positive emotions, as the author says, have many benefits and are pivotal in our ability to learn.

#### Gamification

The prototype presented in this paper is a conversion from a game, which, at first glance, does not need extra elements to be perceived as such. However, some aspects of it can be further improved, for example, a leaderboard that, when visible to all players, stimulates healthy competition. Gamification can be defined as making use of elements typically found in a game in a nongaming context to transform every day, uninteresting tasks, into engaging ones, while increasing user activity and retention [31-33].

Typical game elements with extensive use of gamification and with interest in the physical and digital part of the FGTI prototype include the use of points (and point systems) and the existence of levels. According to Zichermann and Cunningham [31], points are a vital element, and they should always be present at any gamified activity, if not in a visible way, at least visible to the activity designer only, so he or she can assess how the users interact with the activity and design appropriate outcomes [34]. When visible, points allow the user to know how close he or she is to his or her goal, and points can thus be highly motivational [34]. Levels, as the name implies, mark something, in this case they mark in-game progress, and they allow players to be aware of where they are, over time, in relation to the game experience. Levels should be logical in terms of level progression, and they should be easy to add to [34]. By further hiding away the test or activity behind a game-like approach, the stakeholders might feel more relaxed and willing to participate [32].

#### Sum of All Parts

An effective TUI, usable in SSD intervention, should allow some degree of simulation and storytelling, as well as the construction of mental models of knowledge. It should also provide some form of social interaction with the artifact and the other players, all in a playful atmosphere [29]. A TUI can provide natural interaction without emphasizing any cognitive effort—a child does not need to learn or understand a set of rules or settings. The perceived focus is on the action executed and what it can represent. A TUI can help gamify speech and language therapy intervention with a child; it can provide an alternative means to promote children’s speech production; it can help parents by being a “fun” homework exercise to do together and can help KTAs by being an activity that can be developed in a group of normal children and children with SSDs. The prototype’s intended use is at the clinic, the kindergarten, or at home. However, there are challenges to overcome. The prototype must offer more than a traditional game. It has to be lightweight and easy to transport while remaining durable to withstand daily use by children. The software component should incorporate options to intervene in several SSD and remain interesting to play with, while sending data to a log that SLTs can consult later.

### Related Work

Some examples of good practices or cases of success can be found in the literature [35-39], but not one is an exact fit in terms of technological requirements, target population, or intervention area of the current project. A total of 3 projects were considered relevant for the conceptualization of FGTI: first, the *table-to-tablet* (*T2T*) intervention materials, designed to be a reliable and valid solution [40] to be used by Portuguese SLTs when treating children with SSD. It has a physical and a digital version, and SLTs can use them interchangeably, but one does not communicate with the other. Second, the *LinguaBytes* materials, from the Netherlands [41], comprises a full set of exercises and varied activities that are mediated by tangible artefacts. The aim of *LinguaBytes* was to be a tangible language learning system for toddlers with some form of motor disability. Third, *Jabberstamp* [42], developed by a team at the MIT Media Lab (Tangible Media Group), is a tool that allows children to add sound to their drawings, collages, or paintings, enabling them to communicate more effectively before developing or mastering writing skills.

### Relevance and Motivation of the Fishing Game Tangible Interface Prototype

It can be argued that the traditional fishing game is already engaging and in use in intervention by SLTs, similar to other traditional games, such as Bingo or wooden blocks, with the alphabet written on them or animals [43,44]. However, the TUI artefact can present additional advantages:

- It allows the customization of sprites (both the avatar representing the player and the fish). This customization stimulates user engagement and makes each game unique.
- It facilitates the process of preparing the session (SLTs, parents, or KTAs).: The system will set up almost everything.

It eases the burden of certain game-related tasks, such as keeping and updating a score or showing who is winning on a leaderboard. In addition, the software can introduce extra challenges, bonuses, and “power-ups.”

It allows the introduction of extra gamification elements.

It affords extra motivation by presenting a game in a new format that is flexible and allows for uniqueness in each play, potentiating children’s preferences toward digital games [42], while retaining the physical traits.

It can also ease the postintervention process. SLTs do not need to record any data from the session, as a “log” file will be created for them, with all the relevant information needed (player’s names, age, intervention time, and what were the answers of all children). This file can (ideally) be accessed through a Web-based software or emailed to the SLTs.

The prototype, at its core, is the traditional fishing game that so many know. Conceptually speaking, the users can expect to find the same organization, functionalities, and set of rules. As such, previous experiences with a traditional fishing game will allow players to seamlessly use the prototype with just a very quick explanation of some components and their functionalities.

**Figure 1:**
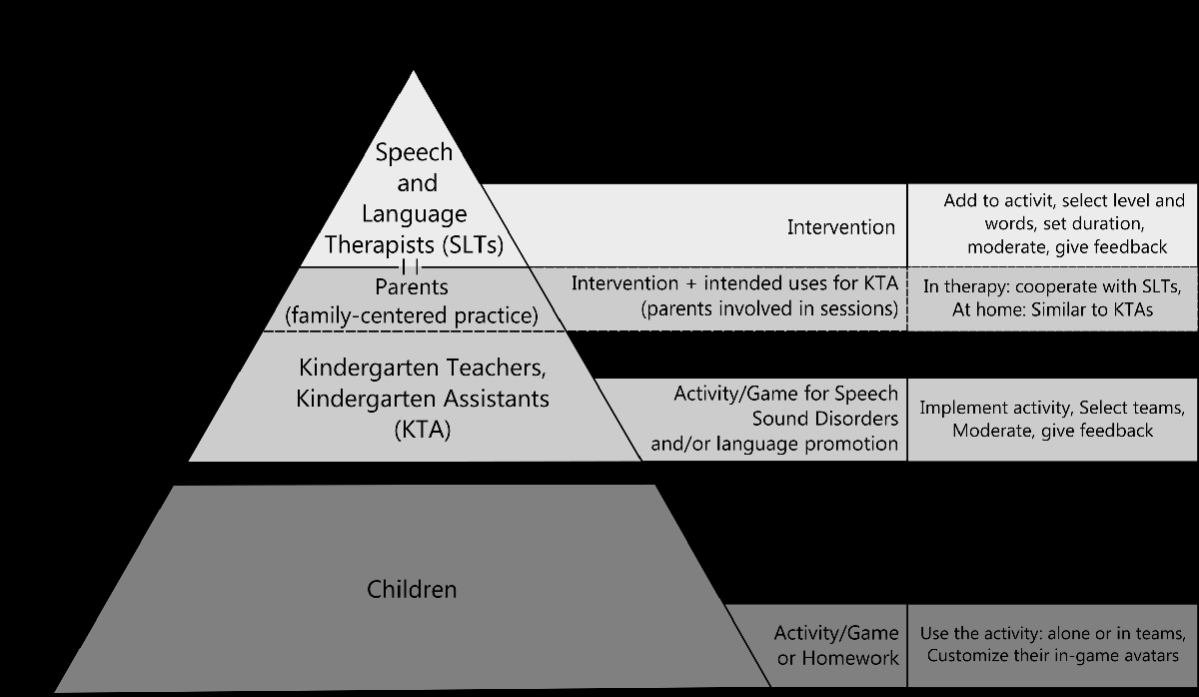
Types of users, their permissions and possible actions within the prototype.

The prototype aimed to be innovative and solve a real-world issue, involving different participants, with diverse roles, as can be seen in Figure 1.

Several design iterations result from this richness in feedback, different uses, and perspectives, because of an encouraged participatory culture (consumer/player active in coproduction) [45]. This constant iteration and evaluation [46] are a trademark of design-based research (DBR). DBR is capable of producing 2 different and nonexclusive outcomes [47]: theoretical (this paper) and practical (the FGTI).

## Methods

### Design-Based Research

DBR allows the researcher to be involved in a way that he or she may glimpse unexpected uses or interactions with the prototype, causing a need to alter it or re-assess the target users, what they do and their needs. To reach the test phase, the physical prototype went through several revisions, always analyzed by SLTs and other project participants, and reworked accordingly. The software part of the prototype (both the game and the web app) was equally revised and improved. To gather data (qualitative and quantitative) from the designated users, in a real-life scenario, an ethnographic approach was used, and a user exploratory test was conducted regarding prototype use. These data were collected through observation and a questionnaire.

### Sample Definition

The sample for the exploratory test comprised 10 expert users, with ages ranging from 4 to 55 years. The parents’ informed consent was procured, in agreement with the World Medical Association’s Declaration of Helsinki regarding human experimentation. Parents also received a document, briefly explaining the test. In addition, ethical permission was obtained from an independent ethics committee (Comissão de Ética da Unidade Investigação em Ciências da Saúde – Enfermagem da Escola Superior de Enfermagem de Coimbra, Coimbra, Portugal), process number P159-05/2013.

The sample can be further clustered into 3 subgroups as follows:

- Children: A total of 6 children, with ages ranging from 4 to 6 years. This group had 2 boys and 4 girls, all speakers with normal development, – no SSD.
- Speech and language therapists: A total of 2 SLTs, with ages ranging from 42 to 54 years. Both are also lecturers at the School of Health Sciences (ESSUA), University of Aveiro, Portugal.
- Kindergarten teachers and assistants: One kindergarten teacher, aged 55 years, and 1 one kindergarten teaching assistant, aged 51 years.

This sample allowed testing of a variety of situations, namely speech and language therapy intervention, children’s use of the activity as a game (group activity), and KTAs with children. The only missing element(s) from the expected users were parents.

### Data Gathering

The technique used was direct observation, although the instruments for data gathering were a form (qualitative data) and a semistructured questionnaire (which allowed the collection of both qualitative and quantitative data). In the creation of the form as an element to annotate the observations, great care was taken in not only dividing the observable actions but also in transforming and categorizing the observable world into interpretable and observable data.

### Exploratory Test: Observation Form and Open Questions

**Figure 2:**
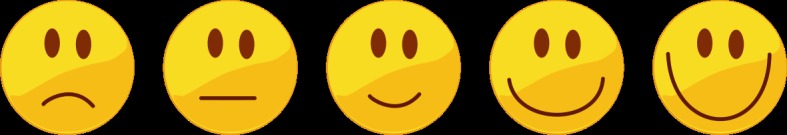
One of the Smileyometers used.

To carry out an effective, direct, and nonintervening observation, especially of an activity involving children and a certain amount of play, a simple and easy-to-complete form was created. A set of open and closed questions, using a visual Likert like scale (a Smileyometer as shown in Figure 2) [48], was also prepared to be used at the end of the test, with the target users (children).

Owing to the variety in the sample and to the constraints an observer may face, a form was prepared to address all scenarios in a single 2-sided A4 page. It was up to the observer to know what he or she was observing and where to annotate it. Both the form and questionnaire used a unique ID for the person observed interacting with the prototype, the type of user he or she was, age, duration of the session, and date.

The form was divided into dimensions or broad areas with clearly defined parameters, to mark as observed (“yes” or “no”), as well as an area reserved to take some quick notes. Those dimensions were as follows:

- Game/Prototype Usability: Parameters regarding the ability to identify the game, its objectives, and components.
- Game/Prototype (Physical) Characteristics: Parameters revolved around the materials, colors (or lack of), robustness, and feedback from the game.
- Gamification: Parameters regarding the desire to play more, if players know when their turn to play is and their score.

The questionnaire was also set on an A4 page, and the observer would choose what to ask and to whom. It was divided into 2 parts, 1 part aimed at the children and 1 part aimed at the SLTs and KTAs. The part aimed at the children had 2 questions, with 2 Smileyometer scales—1 with 5 smiles representing values 1 to 5 for the question “Have you enjoyed playing this game?” The other used 3 smiles, representing values 1 to 3 for the question “Would you play this game again?” The remaining questions were open questions. The last part also had a set of open questions, used to conduct an informal guided interview.

#### Location and Setting Up

The test took place in a kindergarten near the University of Aveiro in Portugal. This setting allowed having, in the same place, 3 of the 5 intended users: the children, the kindergarten teachers and the kindergarten teaching assistants. The fourth intended users, the SLTs, joined the group at the kindergarten. This is also coherent with some SLTs’ intervention locations—kindergartens and schools—allowing the observer to be in the intervention environment instead of a laboratory.

The test was held in the kindergarten library, as it is a quiet and spacious room with plenty of natural light. The prototype was prepared; its contents were set up, and the fish basket was connected to the laptop. A set of printed game rules was available, and the SLTs and KTAs were encouraged to read them. A brief explanation was given of how the prototype worked, the role of the components, and functions of the buttons. This explanation was considered to be similar to the instructions one would receive when buying such equipment. The observer prepared the observation forms and the set of questions to ask the users.

#### Four Possible Use Cases Tested

The prototype’s hardware and software limitations constrained its use to 2 participants at a time. Owing to the number of participants on the exploratory test, more than one expected use scenario was tested.

The first scenario tested was the SLT intervention on a child, using the activity. The second was the kindergarten teaching assistant and a child and their interaction with each other and the activity. The third scenario was a group activity—2 children playing the activity, talking with each other about what the other caught, points won, and related subjects. The fourth and final scenario tested was a child and the kindergarten teacher playing and how both interacted. The only untested scenario was that of parent and child playing the activity because of time constraints. This limitation is discussed at the end of this paper.

### Fishing Game Tangible Interface

The design and functionality of the FGTI is addressed in this subsection, as well as a description of its functionalities and needs, as related to SSD. The hardware and software used to build the prototype and game are briefly described, concluding with a synopsis of the ideas that led to the finished FGTI prototype.

#### Functional Design

**Figure 3:**
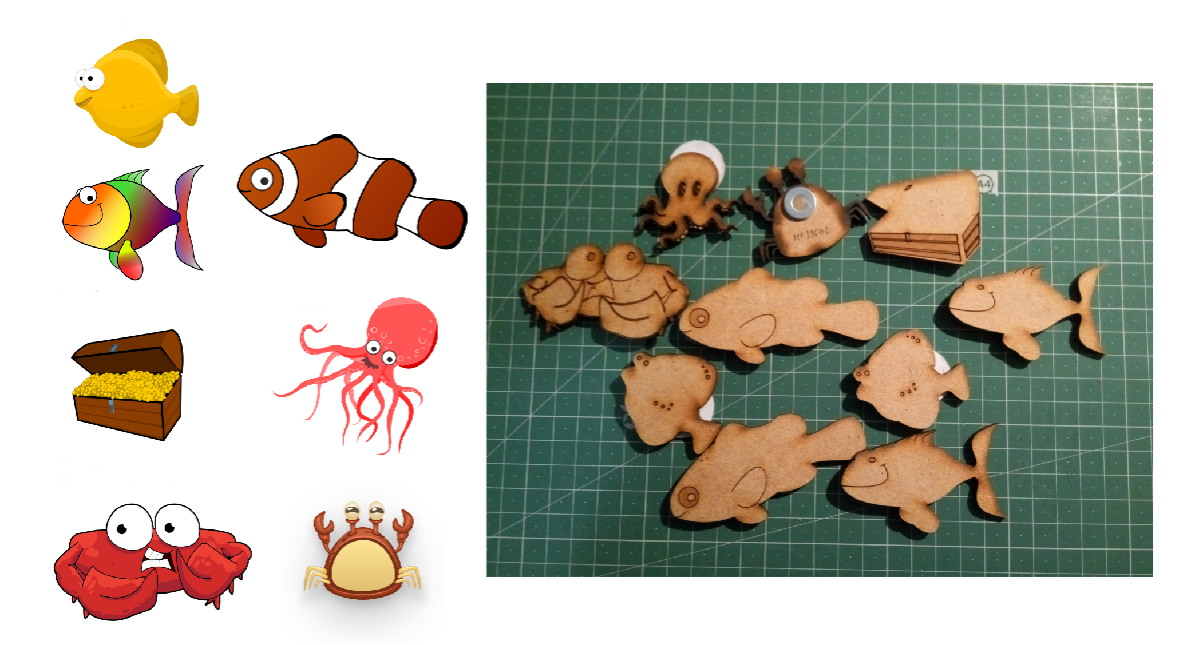
Digital and physical activity assets.

The Fishing Game (or Pond), is a dexterity game, and the rules (which may vary from publisher to publisher or can be determined by the players) are as follows: all sea creatures and treasure chest, seen in Figure 3 in both physical and digital counterparts, go into the pond. The players take turns in attempting to catch a fish with the (magnetic) fishing pole.

Each player has a set number of attempts to try to catch a fish, without looking into the pond, to better emulate a real fishing activity at a lake or ocean. The number of allowable attempts is agreed upon among players at the outset. Each sea creature has a number printed on the back. For older players, the value of the sea creatures is added up to determine the winner. For younger children, the number of caught sea creatures determines the winner.

The TUI prototype rules are broadly the same as described above, with the following exceptions:

- All sea creatures go into the wooden trunk that serves as both a carrying space and the “board” game area.
- Sea creatures will have a certain range of values. For example, a codfish value can go from 5 to 15 points, mimicking the fact that the fish can be a small or a bigger codfish. How much each sea creature is worth will be calculated randomly within a range of values, during the game.

**Figure 4:**
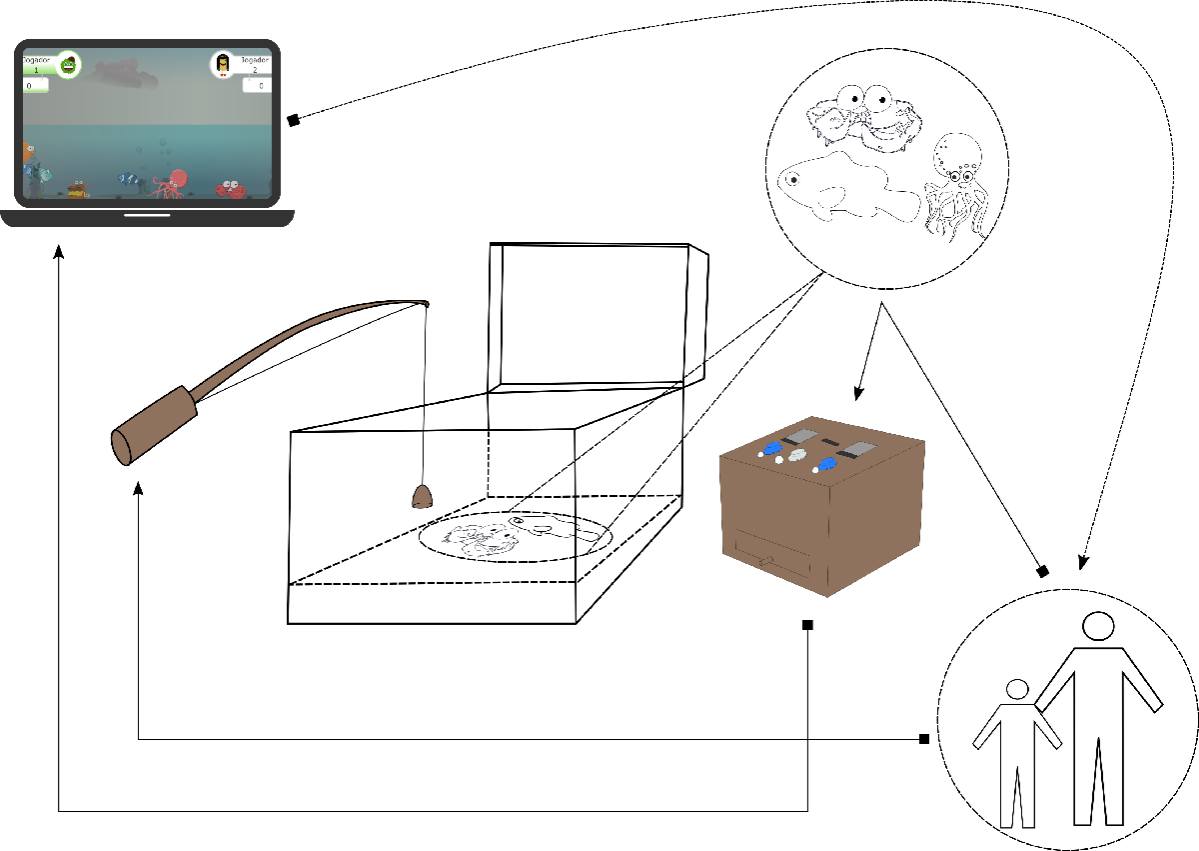
Relevant functional components of the fishing game tangible interface.

In Figure 4, an infographic with the relevant functional components of the FGTI is shown. The solid lines represent interaction, whereas the dashed line represents the visual and auditory cues the player gets from the laptop. The dashed circle and line with the fish represent what is inside the box without perspective skew.

The stakeholders interact with the fishing rod, which in turn interacts (catches) with the fish (see Figure 4). The stakeholders interact with the captured fish by placing them in the fish basket and pressing the necessary buttons to execute the activity (differentiating the word and image) and change player’s turn—the sound cue is repeated at given intervals until the stakeholder acts. The fish basket interacts with the laptop by sending and receiving data according to the fish radio frequency identification (RFID) data or activity moment. The users have constant digital (via screen) or real (via the fish basket, sea creatures, and fishing rod) feedback, expressing the action, points received, or player turn.

**Figure 5:**
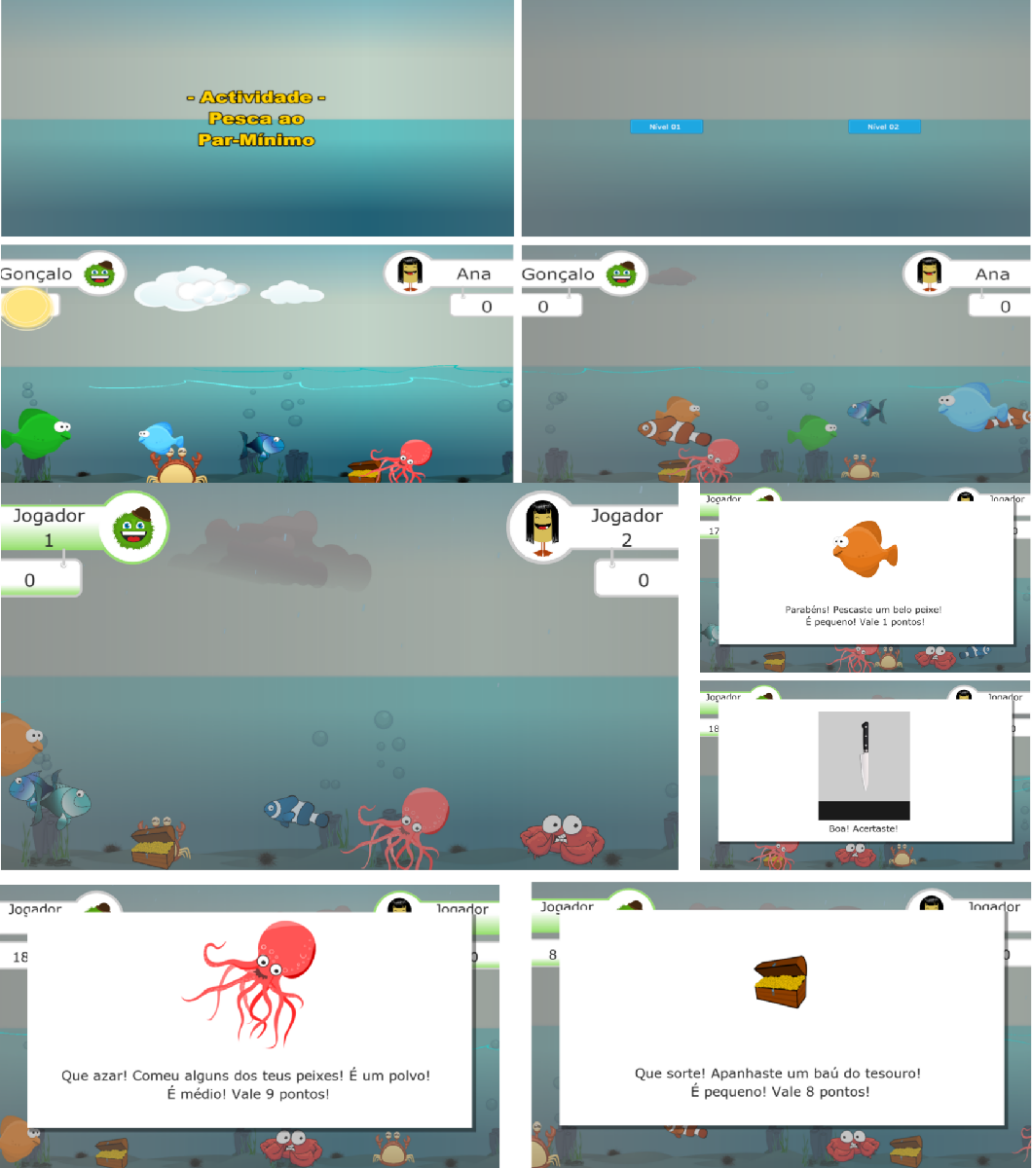
The different screens of the activity.

The activity screens are shown in Figure 5. The stakeholders are greeted by the name of the activity (top left) and have a chance to select the level or have it chosen by the SLTs (top right). Immediately below these images, there is a representation of the visual differences between levels (calm and sunny sea for level 1 and a stormy sea for level 2). The sound effects also add to this atmosphere. The remaining images exemplify the in-game screens, the different catches, and associated messages.

**Figure 6:**
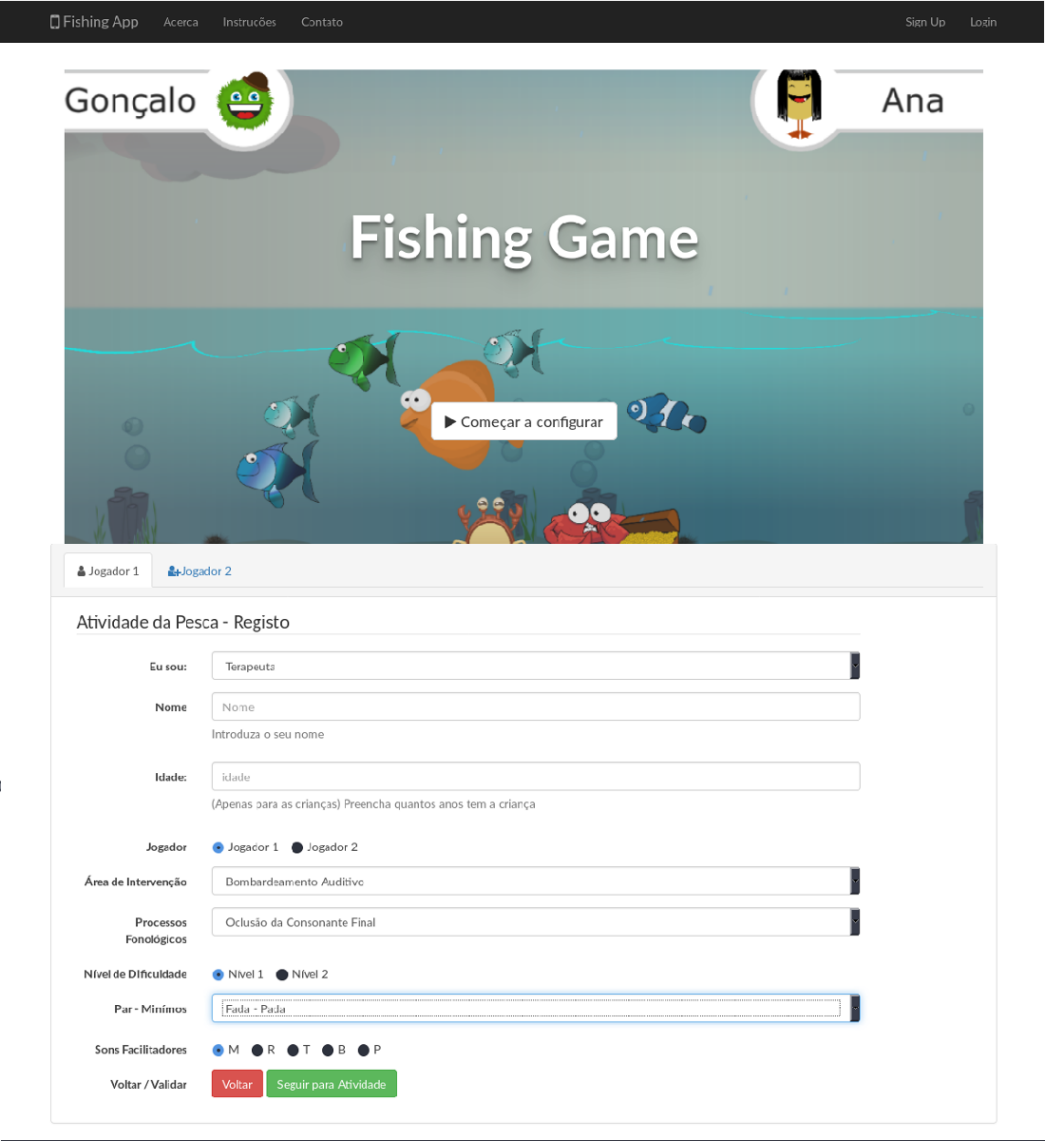
Web companion screen.

The FGTI prototype, with all its components, the activity software, and the Web-based app, seen below in Figure 6, were developed to be as close as possible to the traditional game and to use the rules described above. In the case of the website or app, it was developed to act as a possible replacement for the setting up of both the game or activity and the clinical file of the child.

### Technical Requirements

**Figure 7:**
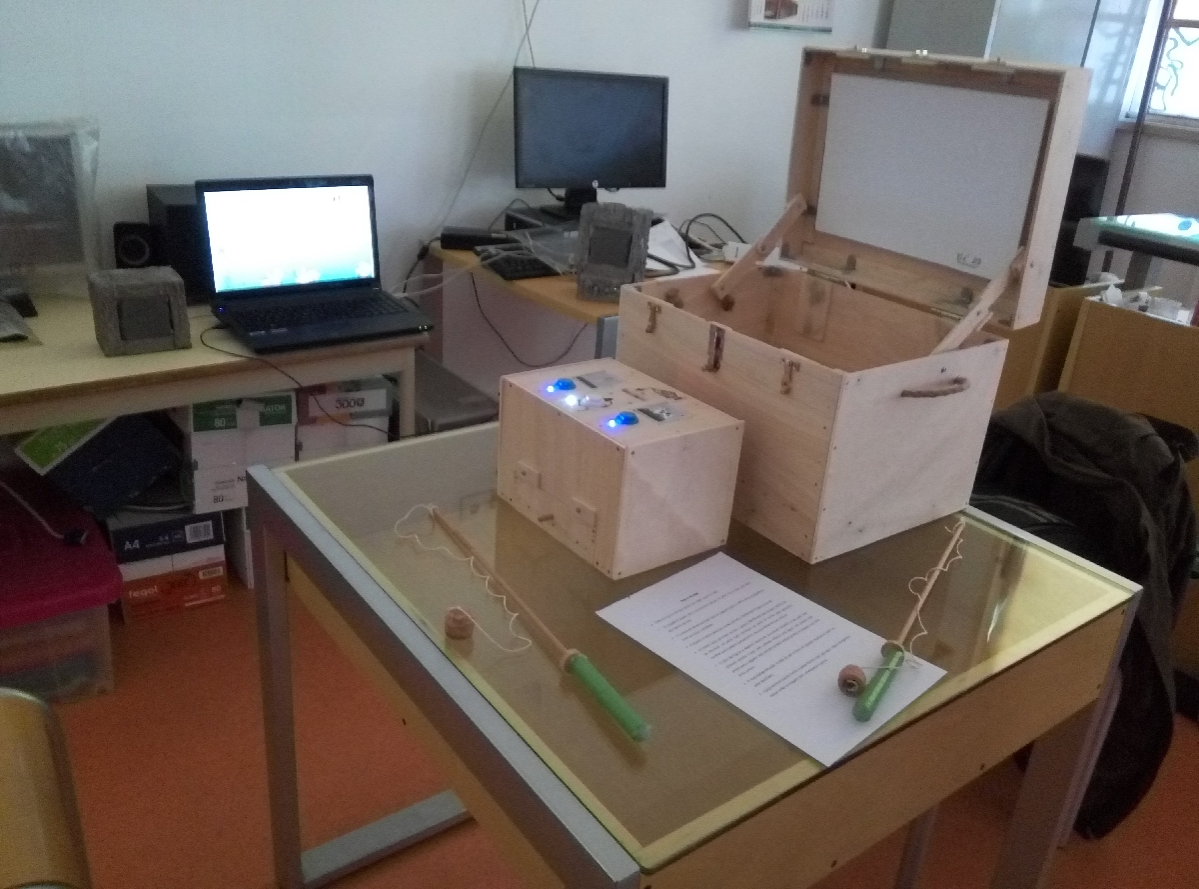
The prototype ready to go.

The FGTI was designed to be self-contained, low cost, and easy to replicate, with embedded physical computing capabilities. Figure 7 shows the complete prototype, assembled and ready to be tested. It was planned to be easy to transport, install, and use “as is.” The only requirement is the presence of an electrical outlet.

#### Hardware System Architecture

**Figure 8:**
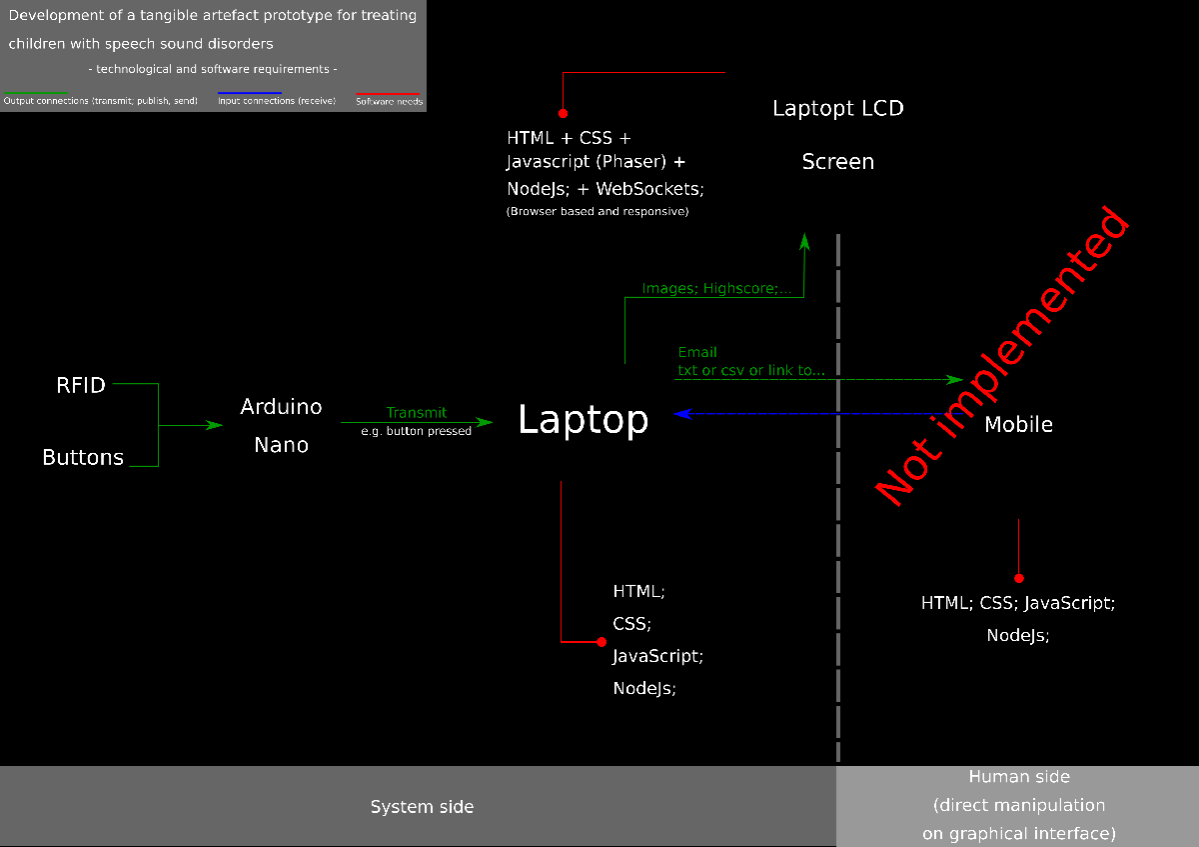
Technological and software requirements diagram.

Owing to time and budget constraints, the liquid crystal display (LCD) screen was not placed in the wooden trunk lid. In addition, a Raspberry Pi approach was discarded for the same reasons, and a laptop (as the prototype “control center”) and its screen (for display purposes) were used instead, as depicted in the block diagram in Figure 8. RFID tags glued on the sea creatures were used, and an RFID reader (RDM6300 125 KHz) communicated with an Arduino via a transmission pin, with a maximum effective (reading) distance of up to 50 mm, taking less than 100 ms to decode the tag or card. An external antenna was placed around the fishing basket slit to read the sea creatures’ RFID tags.

#### Software

The development of this prototype (activity and website or app) involved several programming languages and libraries, and various pieces of software (or scripts) were created.

The activity was coded using a mixture of HTML, cascading style sheets (CSS), and JavaScript, with the Phaser Framework (available at phaser.io.). Phaser is a JavaScript framework for 2D game development in mobile and desktop environments, also ideal for the prototype goals [49]. JavaScript Object Notation (JSON) was used to store several values and properties attributed to each sea creature, for example, name, value (range of), or the sound it represents. To make the activity more scalable and readier to deal with real-time use and requests, Node.JS was also used. Node.JS can use and run a JavaScript server side (traditionally an area for other languages, such as Hypertext Preprocessor or PHP). With Node.JS and its node package manager, several packages were installed to allow bidirectional communication between the activity and the Arduino with the server, via WebSockets (available at socket.io) plus node-serialport (available at serialport.io), or to render the HTML code in the browser through Node (express.js; available at expressjs.com). This installation implied the creation of a simple server-side and client-side set of files to handle requests and responses. The website or app companion used almost all of the previously mentioned languages and technologies, with some exceptions: The Phaser framework was not used; Node.JS was used, but with different packages installed; HTML and CSS played a larger role; Bootstrap (available at getbootstrap.com) was heavily used. Bootstrap is a front-end library that allows one to quickly build or prototype responsive (ie, which can adapt to any screen dimension) websites or apps [50]. The website or app companion can be transformed into a mobile app installer package, with software like Apache Cordova (available at cordova.apache.org). Cordova wraps the code into a native container that can access the mobile device’s native functions (such as the accelerometer) and several different platforms, enabling Web-based software to deploy in any device (platform agnostic) [51]. The software on the Arduino was coded using the Arduino programming language. A specific library was also used to integrate the RDM6300 RFID Reader. Figure 8 presents a diagram of the technological and software requirements, as well as the parts comprising the prototype.

### Prototype Development

The prototype comprised the following parts: wooden trunk, sea creatures, fishing poles, fishing basket, and speakers. Some elements of the prototype, because of their importance, role, or particular challenge, required more than one revision.

#### Wooden Trunk

The design of the wooden trunk prototype, similar to all the elements in the fish game, was based on a maritime theme; therefore, the idea was that it should mimic an old treasure chest. That design was reflected not only in the shape (no round top lid) but also in the choice of ironmongery and straps. It encloses all the components used in the activity, except for a laptop. This laptop is needed to power the Arduino Nano and electronics and to display the digital representation of the fishing activity.

#### Sea Creatures and Treasure Chest

In this prototype, sea creatures have an extra dimension (they are not just an object with a value assigned to it or being counted as one more fish caught by the player) because of their RFID tag. This tag gives users access to as much information as needed, included in the JSON file, read during the activity. Sea creatures and the treasure chest were revised once. Similar to the treasure chest, during the build and actual use, some notes were taken on possible improvements. A total of 9 different sea creatures (6 fish, 2 crabs, and 1 octopus) and a treasure chest were created. They were then laser cut, and the result is shown in Figure 3.

#### Fishing Basket

**Figure 9:**
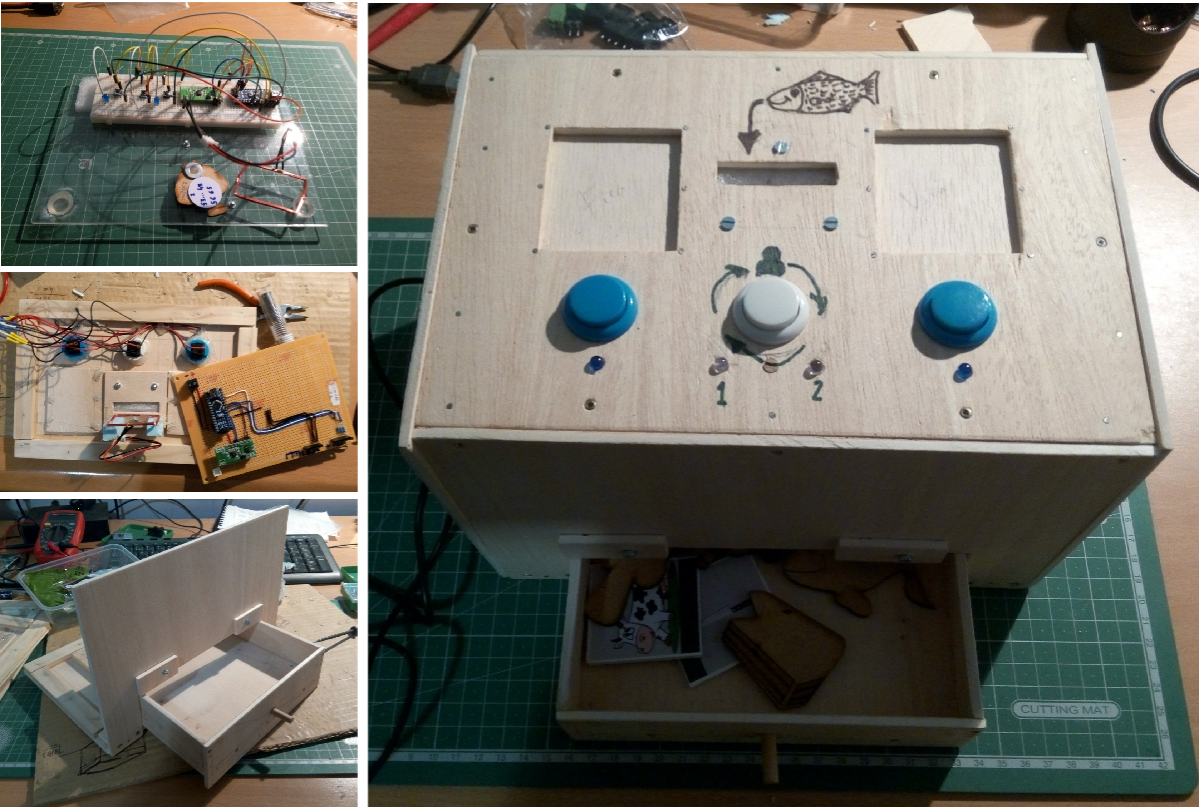
The final iteration of the fish basket, from prototype (top-left corner) to the final result.

The fishing basket, shown in Figure 9, was the prototype element that took the longest to build and develop, mainly because of the fact that it is the most complete and the one closest to the initial idea of how it should be and behave. Owing to its importance and central stage role, it was the driver of several design, technical and material changes. A consideration was always taken as a decisive factor: the end result had to be cheap and easy for any hobbyist to develop.

#### Speaker Rocks and Fishing Poles

The speakers were initially conceived as being encased in the wooden trunk lid, close to the LCD. With a reduction in size of the wooden trunk, the position of the speakers had to be reconsidered. To keep the maritime theme, the idea was to turn the speakers into a rock-like element, similar to those found around piers.

The fishing poles were revised twice (ie, they were built during the initial construction of the wooden trunk and the wooden trunk revision.02). Despite the several ideas resulting from the initial brainstorming sessions, time-related constraints led to a pragmatic and simple approach. The ideas for a more advanced fishing pole were collected to be used in a future iteration of the game.

## Results

### Overview

In this section, the results obtained in the exploratory test are presented and appraised. Some items were not possible to observe, or it was not possible to overhear comments about them among the users. However, they were kept in this section (see tables in the following subsections) because of 2 main reasons: first, being an exploratory test, done to ascertain the pertinence of further studies [52], the authors felt that its absence would skew the test results, and second, as some unobservable items were expected to elicit some sort of feedback (eg, the lack of color on the figures), and as such, the absence of feedback was considered relevant.

### Observation Form

The observation form was divided into 3 dimensions: prototype or activity usability, prototype or activity characteristics, and gamification. It was used for direct observation, without any intervention during the activity. The observer strived to annotate all the interactions and relevant exchanges of commentaries. In the subsections below (in table format), we will present the feedback collected from every area and parameter.

#### Prototype Usability

This dimension explored parameters regarding the users’ ability to identify the game, its objectives, and components. Results are shown in Table 1.

**Table 1:** Prototype usability observation parameters.

Observation parameters	Observation results
Was the player able to identify and recognize the game and its objectives?	A total of 2 out of the 6 children tested immediately identified the game. The others were not vocal enough to demonstrate whether if they knew what they were playing, and the SLTs^a^ (who remained present for the whole duration of the test) or the KTAs^b^ identified the game for them. They all knew the “classic” objectives and assumed that the novel elements (treasure chest, octopus, and crabs) had a similar value and role as the fish. Only 1 player and 1 Speech and Language Therapist noticed that the unusual elements had different score behaviors. A player even said that she liked this game more than the classic that she had at home: “The one I have at home is a blanket and we can stand on it and fish with our hands. But this one is more fun!” – [CT, aged 6 years]. When asked why, she replied that this had a larger variety of sea creatures and some stole points (this child caught an octopus and noticed that it had halved her score). She went on to say to the Kindergarten Teacher that she should buy this game for their classroom.
Was the player able to identify the game components (sea creatures, fishing poles, and wooden trunk)?	All participants were able to correctly identify the game components and even discriminated the sea creatures (saying that one was an octopus, another a crab, etc).
Was the player able to identify the game elements and its function (buttons and slit)?	As none of the children were able to provide a viable answer, the SLTs and the KTAs role -played with them and helped them when they first caught a fish or when they needed to change players or choose a different set of words (minimal -pairs). From that moment on, the game elements and its functions were a learned behavior. The test that involved 2 children playing against each other was also mediated by an SLT; therefore, the same explanations/roleplays were present.
Were the game elements timings correct?	Unfortunately, it was not possible to register this parameter. The children were having such fun while using the prototype and the SLTs and KTAs were so involved with them that none seemed to notice (or care about) the timings needed for a fish to be recognized or for the word to appear. Further testing is required to understand whether the timings are correct. One cannot assume that the stakeholders will be this engaged all the time and must instead assume that the novelty of the situation and the fact that it was a “one-off” test made stakeholders unaware of the timings.
Did the player know when it was his or her turn to play?	Regarding the entire sample, 9 out of 10 knew when his or her turn to play occurred. However, this does not mean that they took the correct steps to play or pass their turn. They all knew that after player *X* would be their turn. But player *X* would usually forget to press the button to change player, or the next player would fail to realize the player number mismatch and correct the situation by pressing the button. This would result in points being given to the wrong player.

^a^SLTs: speech and language therapists.

^b^KTAs: kindergarten teachers and assistants.

#### Prototype (Physical) Characteristics

Parameters in this dimension are those regarding the game components, their physical characteristics, and whether they served their intended purpose. Many of these parameters (see Table 2) were observable only in the children. This limitation was because of the brief explanation given to the SLTs and KTAs, which made them aware of the uses and whys of many of the physical characteristics.

#### Gamification

In this dimension, the parameters observed were those regarding the desire to play more, whether the player knows if or when is his or her turn to play and his or her score. Results are presented in Table 3.

**Table 2:** Prototype physical characteristics observation parameters.

Observation parameters	Observation results
Were the game and its elements adequate?	No comments from the users were registered.
Was the color of the game elements adequate?	There were no comments about the (lack of) color of the physical components of the activity, and little attention was paid to the on-screen elements.
Do the materials used to build the prototype invite the handling of it?	No comments were registered. The observer marked the parameter to ask in the guided conversation with the SLTs^a^ and KTAs^b^.
Was the prototype considered robust?	No comments were registered. The observer marked the parameter to ask in the guided conversation with the SLTs and KTAs.
Was the feedback throughout the activity efficient?	A total of 2 out of the 6 children waited for the feedback (eg, the audio feedback of the word after inserting the sea creatures into the slit) or were aware of it.
Did the physical constraints serve their purpose?	This was observable in 3 out of the 6 children. It was more apparent regarding the slit and its use.
Was the mapping of the buttons and their actions consistent and correctly perceived? Were they used during the activity?	A total of 4 out of the 6 children correctly and consistently used the buttons when they were supposed to and to the desired end.

^a^SLTs: speech and language therapists.

^b^KTAs: kindergarten teachers and assistants.

**Table 3:** Prototype gamification observation parameters.

Observation parameters	Observation results
Were the participants able and willing to play or participate until the end?	All the participants were involved until the end, showing great interest and willingness. Some (children included) even wanted to know details about the study.
Were the participants willing to play more?	A total of 4 out of 6 children asked whether they could play more.
Were the participants aware of their score, at any given time?	A total of 3 children knew their score, 1 child was not aware of it, and for the other 2 children, this was unknown. Despite knowing the score and being quite attentive to the value of each captured sea creature, the children did not seem to have a clear notion of who was winning. They looked at their points on the screen and would say, “I have x points!” but nothing more. When the activity ended, children would often ask who had won.

### Open Questions Questionnaire

The open questions questionnaire was divided into 2 sections: 1 section for children and another section for SLTs and KTAs. Below, we discuss the results and answers obtained in both sections.

#### Children

Children were asked 4 questions, 2 of which using Smileyometers [48], with 2 scales: 1 scale had 5 smiles; therefore, values would range from 1 to 5, whereas the other scale had 3 smiles, with values ranging from 1 to 3. Children being our target users, their feedback was very important; therefore, the observer was very attentive to what they said about the activity. The *first question* was whether the participant had enjoyed playing the activity. The Smileyometer scale was explained to the children in the following manner:

You see, the first smile is sad. He has not enjoyed playing this game. The second smile is –well,...I really do not care about it. The third smile liked it a bit. He could play more...or not! The fourth smile is happy, he enjoyed playing the game. The fifth smile is really really happy because he enjoyed it a lot.

This was said while pointing to each smile in turn. The child was then asked to choose the smile that depicted his or her feeling toward the game he or she had just played.

A total of 5 out of 6 children (83%) picked the happiest smile, which had a value of 5, and just 1 child chose the third smile, which had a value of 3. That is, more children enjoyed playing the activity than not.

The *second question* concerned what feature the stakeholders enjoyed the most. Despite this being an open question (ie, with a broad range of possible answers), most of the children mentioned similar aspects as the most enjoyable. A total of 4 children mentioned that they enjoyed capturing the sea creatures. One child remarked that doing so was like going fishing (actual fishing) with her father. A total of 3 children enjoyed pressing the buttons, and 2 children enjoyed placing the sea creatures in the slit. Some additional comments included that they had enjoyed the fishing pole itself, the crab, the octopus, the chance to play the game, and the variety of sea creatures, as contrasted with the original game using “just” fish.

The *third question* probed what feature the stakeholders enjoyed the least. This was also an open question. A total of 3 children did not identify anything they disliked. The remaining 3 children reported different aspects: 1 child disliked catching the crab as it stole points; another disliked pressing the buttons, explaining, “...you press the button and that’s it!” The third child disliked watching the game on the PC, preferring instead to play it physically. The gameplay was not fully understood by the stakeholders. Many were not aware that catching a crab or an octopus decreases a player’s points. Similarly, many were not aware that answering correctly (pressing the buttons) would double their score. To overcome this issue, a better visual representation of the elements, with both auditory and physical cues, must be implemented on the existing FGTI components or components yet to be developed.

The *fourth question* regarded whether the stakeholder would play again. This was a question made while showing a Smileyometer with 3 values. The scale was explained to the children in the following manner:

Ok we have more smiles. You see, the first smile is sad. He does not want to play again. The second smile is again indifferent; he really does not care if he plays or not. The third smile is so happy he cannot wait to play again.

All children picked the happiest smile (ie, all children wanted to play again).

#### Speech and Language Therapists and Kindergarten Teachers

In relation to the SLTs and KTAs, the open questions were introduced in an informal talk that occurred after the testing of all the children and after them having had a chance to try out the activity themselves. This talk took the form of a fluid conversation, with moments that resembled a brainstorm session. The observer mainly listened, offering his comments occasionally.

The *first question* was whether they would change anything in the concept or prototype. Some said they would not change anything, that it was interesting to see this transformation of a classic game and that it was nice to have the auditory feedback when something was inserted into the slit. Others had more, sometimes divergent, opinions. An SLT said that given children’s (and their own) difficulty remembering to press the button to change players, this function could be automated. This suggestion prompted an immediate response from the other SLT, who said that some children had mentioned that they enjoyed pressing the buttons; therefore, this change in button press should be maintained. What this action needed was more feedback or to be made more visible, she suggested.

The *second question* concerned whether they would change anything in the game or activity. The Kindergarten Teacher said that she would not change anything in the game, as it is so similar to the “original” game that no explanations are needed. However, she felt that the way the score was being handled lacked more and better feedback for the player. This feedback would, in her opinion, increase the competition and game aspect of the activity among the users.

The SLTs both enjoyed the game. They would add more sea creatures, so you can have more auditory stimuli, as at the moment, there are only 3 sea creatures per player: they suggested 6 to 8 sea creatures per player. With larger number of sea creatures, they immediately added that the agents of distraction (the crab and octopus) numbers should also increase to maintain proportion and level of challenge. A sort of “superclass” sea creature, capable of shifting the entire game for all players, was suggested. The way the auditory stimulus was working was also remarked as faulty. Children should pick the sea creature, insert it, and listen to the word (in loop if needed), and only when this action is completed, should they see the image. What was happening was that the image would come up and allow the children to have a visual (not auditory) cue. This feature will be revised in future versions of the prototype.

The *third question* enquired as to how they would expand the prototype if they could. The kindergarten teacher mentioned that she works with bilingual children; furthermore, as a second language is taught at a young age, she then suggested the prototype should be multilingual. The SLTs would like the prototype to have more uses and not be just the game of fishing. They suggested to give names to the sea creatures to make children repeat those names, allowing sound production stimulation. Another approach to extend the game’s functionality would be to use more game scenarios that the adult could choose from. Some sea creatures with some word values associated with them could be tossed into the pond for children to capture. When captured and inserted into the slit, the word would be unveiled, and the children would be encouraged to use it to construct a story.

The *fourth question* was a preamble to the fifth and sixth questions, depending on the answer. The SLTs and KTAs were asked whether they would like to see more games made into TUI. If they answered “yes,” they would go on to question 5—“If yes, which games?”—if not, they would go to question number 6: “If not, why?” They all answered, “yes.”

The *fifth question* was which games they would like to see made into a TUI artifact. The answers were as follows: Tic-Tac-Toe, Snakes and Ladders, Bingo, and an indirect identification game similar to What am I? These can all be possible stand-alone development avenues or add-ons to the existing TUI artifact, adding extra elements and adjusting others, along with the software binding them all.

Questions from the observation form, which were asked during the conversation, as it was deemed more productive, were about the robustness of the prototype and the adequacy of materials and how appealing it was to be used. Both KTAs and SLTs alike said the prototype was robust enough if the activity was used under (adult) supervision. Left unattended, it was robust, but accidents do happen. Regarding the kind of material and its appeal, they all agreed that wood is used quite often and that children are used to seeing it in other toys and enjoy using them. They mentioned that some parts could use other materials to give extra-sensory input that may foster immersion in the activity.

### Speech and Language Therapists and the App

Both SLTs were shown the partially developed hybrid app—hybrid apps are web (browser based) apps, developed using HTML, CSS, and JavaScript, which are then wrapped in a layer that allows interaction with smartphone and tablet hardware and software, independent of code or language. This app will allow one to prepare sessions remotely and provide the visible front of a back-office area where SLTs can keep and generate logs or visual representations (ie, graphics and other visual forms to represent information) from sessions and target users. SLTs can log in to access a roster of target users or add a new target user. They can choose an area of intervention, a set of exercises, what words or facilitator sounds to use, and other functionalities. This Web-based app can even be used by KTAs or parents, which will be able to access and set different parts of the application. Their feedback was collected and saved for a future implementation.

## Discussion

Although it is true that in speech and language therapy, the sound, visual cues from the SLT, and speech production trials are essential, the main purpose of this game was not to replace an SLT but rather to provide a tool, capable of being used by SLTs, parents, or kindergarten staff, with a focus on hearing discrimination instead of articulation. It was felt that if a child struggles to produce a sound, the adult using the game can offer guidance, albeit without the know-how of an SLT. This particular game focused on a single minimal-pairs exercise, which comprises differentiating similar sounding words. Those words or “sound cues” were played until the user decided to press button A or B (associated with an image that corresponded to one of the sound cues). The SLT present in the room, if any, can choose to say the word to show the articulation, if needed.

By using an ethnographic approach and a single observer, present but not participating with any form of help besides the setting up, the impact of the observer presence was kept to a minimum. However, a known risk is that the observer is not entirely unbiased and his/her presence will always have an impact. In addition, the inability to generalize from the data gathered, the sample size and the analytical transparency (the observer was also responsible for coding the data) are points that will have to be addressed in future research, by the use of complementary tools to gather data. Despite these negative points, the richness of the data surmised in location allows for a better insight on the users’ reaction and relation with technology and their natural environments [53,54].

The analysis of quantitative and qualitative data revealed that the prototype addresses the existing needs of SLTs and KTAs and that 5 out of 6 (83%) children enjoyed the activity. Results also revealed a high replay value, with all children saying they would play more. In the following section, we discuss the feedback collected and summarize some ideas and areas for improvement.

### Observation Form

The observation form was divided into 3 dimensions: prototype or activity usability, prototype or activity characteristics, and gamification. It was used for direct observation, without any intervention during the activity. The observer strived to annotate all the interactions and relevant exchanges of commentaries. In the subsections below, we will present the feedback, ideas, and areas for improvement, collected from every area and parameter.

#### Prototype Usability

The feedback and perceived recognition, as well as the ease with which the players started playing the game, are valid indicators that the transposition of the traditional game into the FGTI artifact was successful. The added value/interest of the extra sea creatures (octopus and crab) and treasure chest should be noted. However, the fact that the score was different for each object (as well as the on-screen object scale that changed according to the points earned) was not readily seen, and only 2 out of 10 participants noticed it. Better audio and visual feedback are needed in the next iteration to ensure this is an understood behavior, which can transform the gameplay experience. The game components seem to be well designed and help in identifying the game and the activity (fishing) on which the game is based. Some sea creatures can be further developed, as the stakeholders engaged in conversations about fish variety (eg, is it a whitefish or a cod?). Further testing with all the stakeholders is required to understand whether the affordances and design work and whether, within minutes, the stakeholders are ready to use the activity to its fullest. Furthermore, testing is required to understand whether the timings are correct. One cannot assume that the stakeholders will always be highly engaged in each play-through of the game, assuming that the novelty of the situation and the fact that it was a “one-off” test made stakeholders unaware of the timings. Knowing one’s place in a queue, which in this case was his or her turn to play, does not seem dependent (especially in an activity with just 2 players) on any sort of visual or auditory feedback. It is something that the players do and know. However, interacting with a button to signal this change is not an expected behavior, and as such, despite knowing (SLTs and KTAs) or being told (children) and despite the markings above the button, this was generally overlooked and was a source of distress.

#### Prototype Physical Characteristics

Further testing is required to understand whether the dimensions are correct and to determine the importance of color. As the digital part was running on the laptop screen, off-angle relative to the wooden trunk and the main activity area, it may have been perceived as a “secondary” thing to look at. Tighter integration of the display (ie, with the LCD on the wooden trunk lid) may lead to a better understanding and ability to test this parameter. Additional testing and modifications are needed to improve on feedback, as per user suggestions and observation.

On at least 2 occasions, sea creatures were pushed into the slot so fast that the RFID reader was unable to detect them; therefore, this issue also has to be considered.

Several suggestions were given by the SLTs and KTAs to slightly alter the phrases and sounds to give more feedback regarding, for example, the score. A possible reason for half the children being aware of the physical constraints, such as the slit and its use, may relate to their turn in playing the activity. The first one to play used the constraint (and the SLTs/KTAs help) to know what to do with the sea creatures and the slit, whereas the second did it because she or he had watched the first one do it.

The mapping of the buttons and their actions were generally well understood and used. There was some “natural resistance” against using the button to change players; the users seemed to expect it to be automatic. The 2 children who did not seem to be capable of using the buttons did play the activity and enjoyed it. The other player or the SLTs/KTAs helped them with the buttons, and they started using them after a short while.

#### Gamification

All stakeholders enjoyed taking part in the activity. Although some of this (especially regarding children) can be dismissed or looked at, considering the novelty factor, it is encouraging to get this feedback.

More than half stated their desire to play more. The remainder may not have expressed this desire for a number of reasons: for example, the test being run just before the Christmas holidays and children being busy taking part in dance or music rehearsals for the school’s Christmas show; therefore, they wanted to go back to those activities.

The stakeholders were aware of their score, or they at least knew where to look at, on screen. However, most of them were unable to say who had won and by how many points.

### Open Questions Questionnaire

The open questions questionnaire was divided into 2 sections: children and SLTs and KTAs. Below, we discuss the answers obtained in both sections.

#### Children

The majority of children enjoyed playing the activity. Even the child who answered differently did not choose a negative smile or value; therefore, even she would play.

A clear pattern could be observed: Children enjoyed playing the game. The game is varied and rich enough to be attractive and fun; however, the digital part of it went unnoticed. This oversight may arise from the previously mentioned off-angle screen. Further testing with a new FGTI revision (of all its components) is required.

All children wanted to play again. Even considering a factor like “novelty,” the results are encouraging and very positive. What remains unclear and depends on further and more exhaustive testing is whether this willingness to play would be sustained after a number of games played.

#### Speech and Language Therapist and Kindergarten Teachers

A need and desire for tools, such as the FGTI prototype, were clearly observed. The prototype was seen as almost ready for serious field and clinical testing after some small bugs and feedback issues were solved. Both SLTs and KTAs said it was something that they could see themselves using with their children.

### Suggestions and Improvements on the Basis of Feedback Gathered During the Exploratory Test

During the entirety of the exploratory test, the observer was able to collect much input and feedback, some direct, as in the final conversation with SLTs and KTAs, as well as some indirect, overheard during the activity testing. Those suggesting improvements and feedback are addressed in this section. Keeping with the maritime theme, a pirate object/figure could be created. This object would be able to steal all points from all players (in practice, resetting the entire score and doing “tabula rasa” of all progress) and sail away (meaning the pirate would be thrown again into the wooden trunk). This new element would be used to counterbalance the treasure chest element and would increase the challenge factor. In addition, the score could be displayed inside a starfish and could have some sort of animation to increase the visual feedback. A total of 2 sets of scores could be used. One, to reward the better fisherman, would be based on a number of sea creatures caught and how much they are worth. The other score would be to reward the player who answered correctly more times (in practice who did better at word discrimination, in the case of the minimal pairs). This reward would make being attentive and answering correctly worth more to a player. The number of points for each catch should be more evident with the use of auditory cues. In the game’s present iteration, a voice can be heard saying, “You caught a fish. It’s small.” The voice should say, “You caught a fish. It’s small. It’s worth *X* points!” Similarly, the voice should also say, after some interaction from the player, (eg, a successful catch but unable to discriminate the word, catching a crab) the number of points the player has. Furthermore, what the voice says and what is written on screen have slight differences that should be addressed. For example, the voice says (in Portuguese), “Tenta de novo!” *(Try again)* and what appears written is, “Tenta outra vez!” One SLT felt that it would be interesting and would help with the immersion to see a representation of the fishing pole on screen, to have an idea of its (relative) position.

### Conclusions and Future Work

The feedback, the perceived recognition, and the ease with which children started playing the game are valid indicators that the transposition of the traditional game into the TUI artifact was successful. The added interest in the extra sea creatures and treasure chest should be noted. However, the fact that the score differed for each object (as well as the on-screen object scale that changed according to the points earned) was not readily seen, and only 2 of 10 participants noticed it. Better audio and visual feedback is needed in the next iteration to ensure that this is an understood behavior, which can transform the gameplay experience. Several suggestions were given by the SLTs and KTAs to slightly alter the phrases and sounds, to give more feedback regarding, for example, the score. Regarding the SLTs and KTAs, a need and desire for tools, such as the FGTI, were clearly observed. The FGTI and what can be produced with the concept behind it can be extremely modular and versatile. However, certain aspects should be improved to have an even better product. The TUI game of “Pond,” with the interest caused and ease of use, appears to be a suitable tool for the classroom or as an SLT set of intervention tools; however, there should be more tests with users, which would produce more iterations of all aspects of the prototype (physical and software). Owing to time constraints, an important group of stakeholders, the parents, was not tested, but they must be involved, observed, and questioned in future work. They share, with the child, a very important space (home), and they spend special time and form unique bonds; therefore, they possess unheard information, not available to any of the other stakeholders. A multidisciplinary team that included children, SLTs, KTAs, and parents as co-designers would allow the development of an end product that is suitable to respond to each user’s needs and desires. More diverse means of gathering data and analyzing data should be employed to minimize the known flaws of ethnography. The FGTI’s perceived value and the positive impact it may have as a tool for intervention for children with SSD should be further expanded. The monitoring part of the game, capable of producing usable reports for the SLTs, will be implemented in future revisions.

## Abbreviations

CSS: cascading style sheets
DBR: design-based research
FGTI: fishing game tangible interface
JSON: JavaScript Object Notation
KTAs: kindergarten teachers and assistants
LCD: liquid crystal display
RFID: radio frequency identification
SLT: speech and language therapist
SLTs: speech and language therapists
SSD: speech sound disorders
TUI: tangible user interface
